# Perceptual Evaluation of Binaural MVDR-Based Algorithms to Preserve the Interaural Coherence of Diffuse Noise Fields

**DOI:** 10.1177/2331216520919573

**Published:** 2020-04-27

**Authors:** Nico Gößling, Daniel Marquardt, Simon Doclo

**Affiliations:** 1 Department of Medical Physics and Acoustics and Cluster of Excellence Hearing4all, University of Oldenburg; 2 Starkey Hearing Technologies, Eden Prairie, Minnesota, United States

**Keywords:** binaural hearing aids, spatial release from masking, speech enhancement, speech intelligibility, spatial quality

## Abstract

Besides improving speech intelligibility in background noise, another important objective of noise reduction algorithms for binaural hearing devices is preserving the spatial impression for the listener. In this study, we evaluate the performance of several recently proposed noise reduction algorithms based on the binaural minimum-variance-distortionless-response (MVDR) beamformer, which trade-off between noise reduction performance and preservation of the interaural coherence (IC) for diffuse noise fields. Aiming at a perceptually optimized result, this trade-off is determined based on the IC discrimination ability of the human auditory system. The algorithms are evaluated with normal-hearing participants for an anechoic scenario and a reverberant cafeteria scenario, in terms of both speech intelligibility using a matrix sentence test and spatial quality using a MUlti Stimulus test with Hidden Reference and Anchor (MUSHRA). The results show that all the binaural noise reduction algorithms are able to improve speech intelligibility compared with the unprocessed microphone signals, where partially preserving the IC of the diffuse noise field leads to a significant improvement in perceived spatial quality compared with the binaural MVDR beamformer while hardly affecting speech intelligibility.

Noise reduction algorithms for head-mounted assistive listening devices (e.g., hearing aids, hearables, and headsets) are crucial to improve speech intelligibility and speech quality in background noise. For a binaural configuration, consisting of a device on the left and the right ear, noise reduction algorithms that simultaneously use the microphone signals from both devices are promising because the spatial information captured by all microphones can be exploited (Doclo et al., 2018; Hamacher et al., 2008; Wouters et al., 2013). Besides reducing background noise and limiting speech distortion, another important objective of a binaural algorithm is preserving the spatial impression of the acoustical scene for the listener, such that confusions due to a possible mismatch between acoustical and visual information can be avoided. This is achieved by preserving the binaural cues, that is, the interaural level difference (ILD) and the interaural time difference (ITD), of all directional sources (e.g., distinct speakers) and the interaural coherence (IC) of nondirectional sources (e.g., diffuse babble noise).

Binaural cues and IC play a major role in spatial perception, for example, for localizing sources and for determining the spatial width or diffuseness of auditory objects (Blauert, 1997; Bregman, 1994). On the one hand, for a directional source, the spatial perception can be well described by the ITD and ILD cues, while IC is rather a measure for their reliability, especially in reverberant environments (Faller & Merimaa, 2004). Rakerd and Hartmann (2010)showed that the IC is related to ITD sensitivity of the human auditory system. On the other hand, for a diffuse sound field, the spatial perception cannot be described by the ITD and ILD cues, while IC can be used to describe the perceived spatial width or diffuseness (Bradley & Soulodre, 1995; Kurozumi & Ohgushi, 1983; Schroeder et al., 1974).

Furthermore, binaural cues and IC are very important for speech intelligibility due to spatial release from masking (Beutelmann & Brand, 2006; Bronkhorst & Plomp, 1988; Hawley et al., 2004; Lavandier & Culling, 2010; Pastore & Yost, 2017). Bronkhorst and Plomp (1992)reported that for four maskers which were symmetrically placed around the listener, a speech reception threshold (SRT) improvement of 1.9 dB could be achieved compared with when all maskers were placed in front of the listener. For a scenario with one-directional speech source in a diffuse noise field, as considered in this study, an improvement of the SRT up to 3 dB has been reported for binaural hearing compared with monaural hearing (Arweiler & Buchholz, 2011), whereas no such SRT improvement can be observed if the speech source and the noise both come from the same direction (Hawley et al., 2004).

Hence, for a speech source in a diffuse noise field, it is important that binaural noise reduction algorithms preserve the diffuseness (i.e., the IC) of the noise field as much as possible in order to preserve the spatial separation between the speech source and the noise, enabling the listener to exploit the binaural hearing advantage.

Several studies have shown the benefits of binaural processing, that is, processing the microphone signals from both devices simultaneously, compared with bilateral processing, that is, processing the microphone signals from each device separately, in terms of both speech intelligibility improvement and spatial perception (e.g., Best et al., 2015; Cornelis et al., 2012; van den Bogaert et al., 2008, 2009; Völker et al., 2015). To combine noise reduction and binaural cue or IC preservation, two different paradigms are typically adopted (Doclo et al., 2018). In the first paradigm, two microphone signals, that is, one from each device, are filtered with the same (real-valued) spectro-temporal gain, which intrinsically guarantees binaural cue preservation for all sound sources (e.g., Baumgärtel et al., 2015; Bissmeyer & Goldsworthy, 2017; Enzner et al., 2016; Grimm et al., 2009; Kamkar-Parsi & Bouchard, 2011; Lotter & Vary, 2006; Reindl et al., 2013; Wittkop & Hohmann, 2003). In the second paradigm, considered in this study, all available microphone signals from both devices are processed by different (complex-valued) spatial filters. Although the second paradigm allows for a very good noise reduction performance and binaural cue preservation of the speech source, the binaural cues and the IC of the noise are typically distorted. A variety of algorithms have been proposed, aiming at also preserving the binaural cues or the IC of the noise (Aichner et al., 2007; Best et al., 2017; Cornelis et al., 2010; Hadad et al., 2015, 2016; Itturriet & Costa, 2019; Klasen et al., 2007; Koutrouvelis et al., 2017; Marquardt, Hadad, et al., 2015; Marquardt, Hohmann, et al., 2015; Marquardt & Doclo, 2018; Welker et al., 1997).

In this study, we assume one desired directional speech source in a diffuse noise field and focus on binaural noise reduction algorithms based on the well-known minimum-variance-distortionless-response (MVDR) beamformer (Doclo et al., 2015; Van Veen & Buckley, 1988). The binaural MVDR beamformer can be considered a special case of the binaural multichannel Wiener filter (MWF), where only spatial and no spectral filtering is applied (Doclo et al., 2018; Gannot et al., 2017). In the case of a single desired speech source, it was shown that the binaural MVDR beamformer preserves the binaural cues of the speech component but distorts the binaural cues of the diffuse noise component (Cornelis et al., 2010). More precisely, after applying the binaural MVDR beamformer, both output components exhibit the binaural cues of the speech component, such that both components are perceived as coming from the same direction and the binaural hearing advantage cannot be exploited by the auditory system. Aiming at preserving the spatial characteristics of a diffuse noise field, more in particular the IC, several extensions of the binaural MVDR beamformer have been recently proposed. The MVDR-IC (Marquardt, Hohmann, et al., 2015) aims at achieving a desired IC for the diffuse noise component by incorporating an IC preservation term into the MVDR optimization problem. The binaural MVDR beamformer with partial noise estimation (MVDR-N) aims for the output noise component to be equal to a scaled version of the diffuse noise component in the reference microphone signals (Cornelis et al., 2010; Klasen et al., 2007; Marquardt & Doclo, 2018). Both the MVDR-IC and the MVDR-N contain a (frequency-dependent) trade-off parameter, which allows a trade-off between IC preservation of the diffuse noise component and noise reduction performance. Based on the IC discrimination ability of the human auditory system in a diffuse noise field, psycho-acoustically motivated trade-off parameters have been proposed for the MVDR-IC (Marquardt, Hohmann, et al., 2015) and for the MVDR-N (Marquardt & Doclo, 2018).

In this study with normal-hearing participants, we report perceptual comparisons of several binaural MVDR-based noise reduction algorithms in diffuse noise fields. To assess the influence of the trade-off between noise reduction and IC preservation of the diffuse noise component on speech intelligibility and perceived spatial quality, we considered the binaural MVDR beamformer, which maximizes the noise reduction performance but does not preserve the IC of the diffuse noise component, and two extensions (MVDR-IC and MVDR-N), which aim at preserving the IC of the diffuse noise component at the cost of decreased noise reduction performance. For the MVDR-IC and the MVDR-N, we evaluated two psycho-acoustically motivated upper boundaries for the magnitude squared coherence (MSC) of the output noise component. In addition, we considered an (artificial) optimal binaural MVDR beamformer (MVDR-OPT) with perfect IC preservation of the diffuse noise component to assess the upper performance limit of combined noise reduction and perfect IC preservation.

All the beamformer algorithms were evaluated with German-speaking normal-hearing participants for an anechoic scenario and a reverberant cafeteria scenario. Speech intelligibility has been measured using a German matrix sentence test (Wagener, Brand, et al., 1999a, 1999b; Wagener, Kühnel, et al., 1999), while spatial quality has been measured using a procedure similar to the MUlti Stimulus test with Hidden Reference and Anchor (MUSHRA) (International Telecommunications Union-Recommendation BS.1534-1, 2003).

The results show that perfect IC preservation of the diffuse noise component (MVDR-OPT) led to SRT improvements of about 2 dB compared with the MVDR without IC preservation, both for the anechoic and for the reverberant scenario. For the practically feasible extensions of the MVDR, that is, the MVDR-IC and MVDR-N, the results show that partially preserving the IC of the diffuse noise component significantly improved spatial quality compared with the MVDR while only marginally affecting speech intelligibility.

## Relation to Previous Studies

The influence of IC on signal detection, speech intelligibility, and spatial perception has been investigated for several decades. Koenig et al. (1977)investigated the influence of reverberation as a masker on signal detection and reported a masking-level-difference of around 3 dB compared with the reference condition, where the signal and the masker were binaurally in phase. In Koehnke et al. (1986), it was shown that interaural correlation discrimination and binaural detection are closely related by measuring psychometric functions with pure-tone signals and one-third octave noise maskers for several degrees of interaural correlation. This study also reported large differences in binaural performance between participants. Lavandier and Culling (2008)investigated the effect of the direct-to-reverberant ratio on speech intelligibility and reported that less coherent noise, corresponding to a smaller direct-to-reverberant ratio, seemed to be more difficult to cancel out by the auditory system. In terms of spatial perception, Kurozumi and Ohgushi (1983)showed that IC is strongly correlated with perceived width and distance. Furthermore, the study reported that the perceived width of a sound source mainly depends on the absolute value of IC (smaller absolute IC led to wider perception) and that the effect of IC is greater for low frequencies (below 1 kHz). Bradley and Soulodre (1995)later also confirmed in a controlled perceptual study with simulated sound fields in an anechoic room that IC as an objective measure strongly correlates with listener envelopment. Walther and Faller (2013)measured frequency-dependent IC discrimination thresholds in a diffuse noise field to investigate the sensitivity of the human auditory system to IC deviations. The results showed that for a reference IC near 1, small deviations can be perceived, whereas for a reference IC near 0, the deviations must be significantly larger in order to be perceived.

The effect of several binaural noise reduction algorithms on speech intelligibility and spatial perception has been investigated in Cornelis et al. (2012), van den Bogaert et al. (2008, 2009), and Völker et al. (2015), mainly comparing bilateral and binaural algorithms. In van den Bogaert et al. (2009), the effect of bilateral and binaural MWF-based noise reduction algorithms on speech intelligibility in terms of SRT improvement was investigated for a single speech source and up to three-directional noise sources. The results showed that the binaural algorithms outperformed the bilateral algorithms and that partial noise estimation with a fixed trade-off parameter of 0.2 only reduced speech intelligibility in a limited way. For the same set of algorithms, van den Bogaert et al. (2008)investigated the localization error for a single speech source and a directional noise source. The results showed that the binaural MWF preserved localization of the speech source but distorted localization of the directional noise source. Furthermore, the results showed that for a directional noise source, applying partial noise estimation with a fixed trade-off parameter of 0.2 enabled correct localization of both the speech source and the directional noise source. Cornelis et al. (2012)compared realistic implementations of the aforementioned bilateral and binaural MWF-based algorithms in terms of SRT improvement for a single speech source in multitalker noise and in pseudo-cafeteria noise. The results showed that the binaural algorithms consistently outperformed the bilateral algorithms by about 2 dB. In Völker et al. (2015), a bilateral baseline algorithm was compared with fixed and adaptive implementations of the binaural MVDR in terms of SRT improvement for a single speech source in multitalker noise, in realistic cafeteria noise and with a directional noise source. Compared with the unprocessed input, the adaptive and fixed MVDR implementations led to SRT improvements between 3 dB and 4.8 dB, depending on the noise scenario.

Contrary to these previous studies, this study is not about the comparison between bilateral and binaural noise reduction algorithms. Instead, we compare different binaural MVDR-based algorithms in terms of SRT improvement and perceived spatial quality. Also, most previous studies only considered (one or more) directional noise sources and focused on the preservation of binaural cues, that is, ILDs and ITDs, we assume a diffuse noise field and focus on the preservation of IC, and whereas a fixed trade-off parameter was used before for partial noise estimation, we use psycho-acoustically motivated trade-off parameters, as proposed in Marquardt, Hohmann, et al. (2015)and Marquardt and Doclo (2018).

## Methods and Materials

### Binaural Noise Reduction and Cue Preservation Algorithms

In this section, we briefly describe the main design objectives and properties for four binaural MVDR-based algorithms, which have different capabilities in terms of noise reduction and IC preservation of the diffuse noise component. For two of the algorithms, we also describe a psycho-acoustically motivated procedure to determine trade-off parameters that enables a trade-off between noise reduction performance and IC preservation of the diffuse noise component.

#### Algorithm 1: Binaural MVDR Beamformer

The binaural MVDR beamformer (Cornelis et al., 2010; Doclo et al., 2015) minimizes the output power of the noise component in both devices while preserving the desired speech component in the reference microphone signals. In the frequency-domain, the left and right filter vectors of the binaural MVDR are given by (Doclo et al., 2015)

wMVDR,Lω=Γ-1ωaωaHωΓ-1ωaωaL*ω 

wMVDR,Rω=Γ-1ωaωaHωΓ-1ωaωaR*ω 

with ωthe normalized radian frequency, Γthe (time-invariant) spatial coherence matrix of the diffuse noise component, the vector acontaining the anechoic acoustic transfer functions (ATFs) between the speech source and all microphones, $a_{L}$the ATF between the speech source and the left reference microphone and $a_{R}$the ATF between the speech source and the right reference microphone. ${\left(\cdot \right)}^{-1}$denotes inversion, ${\left(\cdot \right)}^{H}$denotes complex conjugate transpose, and ${\left(\cdot \right)}^{*}$denotes complex conjugation.

**Figure 1.**
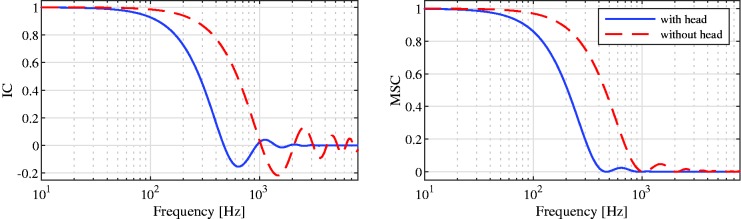
Theoretical IC (left) and Corresponding MSC (right) of a Diffuse Noise Field With and Without Considering the Effect of a Head. IC = interaural coherence; MSC = magnitude squared coherence.

To compute the filter vectors of the binaural MVDR, design choices for the parameters Γand aneed to be made. For a diffuse noise field, Γis typically modeled by assuming a spherically isotropic noise field, for which the spatial coherence between two microphones in free field is equal to sinc(ωd/c), with *d*the intermicrophone distance and *c*the speed of sound (Cron & Sherman, 1962). When the microphones are mounted on a head (e.g., modeled as a sphere), the IC of a diffuse noise field can be physically modeled (Jeub et al., 2011) or approximated using a modified sinc function (Lindevald & Benade, 1986). This is shown in Figure 1, where the theoretical IC and the corresponding MSC of a (spherically isotropic) diffuse noise field with and without including a head between the microphones are depicted. Note that at low frequencies the IC is rather large, whereas at higher frequencies, the IC is typically very small. The anechoic ATFs in amay be simulated or selected from a database with measured ATFs such as Kayser et al. (2009). In this study, it is assumed that the desired speech source is located in front of the listener and the direction of the desired speech source is known.

As shown in Cornelis et al. (2010), the binaural MVDR preserves the binaural cues of the desired speech component but distorts the diffuse noise component, such that after applying the binaural MVDR the binaural cues of both the output speech component and the output noise component are equal to the binaural cues of the input speech component. Hence, at the output of the binaural MVDR, no spatial separation between the desired speech component and the diffuse noise component exists anymore, such that both components are perceived as coming from the same direction and the binaural hearing advantage cannot be exploited by the auditory system.

#### Algorithm 2: Optimal Binaural MVDR Beamformer With Perfect IC Preservation (MVDR-OPT)

To define an upper performance limit for combined noise reduction and IC preservation that enables us to assess the binaural hearing advantage of the participants, we considered an (artificial) optimal processing strategy, denoted as MVDR-OPT. This optimal processing strategy yields the same output signal-to-noise ratio (SNR) as the MVDR but perfectly preserves the IC of the diffuse noise component. The output speech component of the MVDR-OPT in the left and the right device is equal to the output speech component of the MVDR. The output noise component of the MVDR-OPT in the left and the right device is equal to a scaled version of the input (diffuse) noise component in the left and the right reference microphone signals, such that the power of the output noise component is exactly equal to the power of the output noise component of the MVDR. Hence, the MVDR and the MVDR-OPT only differ in terms of the IC of the output noise component but not in terms of output SNR. The output IC of the noise component for the MVDR-OPT is exactly equal to the input IC of the diffuse noise component, whereas the absolute value of the output IC of the noise component for the MVDR is equal to 1.

#### Algorithm 3: Binaural MVDR Beamformer With IC preservation (MVDR-IC)

As the MVDR does not preserve the IC of the diffuse noise component, an extension for diffuse noise fields was proposed in Marquardt, Hohmann, et al. (2015), denoted as MVDR-IC. The MVDR-IC aims at achieving a desired IC for the output noise component by extending the MVDR optimization problem with an IC preservation term. As no closed-form expression is available for the filter vectors of the MVDR-IC, an iterative numerical optimization method needs to be used (Marquardt, Hohmann, et al., 2015).

It was experimentally shown in Marquardt, Hohmann, et al. (2015)and Marquardt and Doclo (2018)that the MVDR-IC almost perfectly preserves the binaural cues of the desired speech component and that a trade-off exists between IC preservation of the diffuse noise component and reduction of the diffuse noise component, which can be controlled by a trade-off parameter.

#### Algorithm 4: Binaural MVDR Beamformer With Partial Noise Estimation (MVDR-N)

Compared with the MVDR-IC, the binaural MVDR beamformer with partial noise estimation (Cornelis et al., 2010; Klasen et al., 2007; Marquardt & Doclo, 2018), denoted as MVDR-N, is a more general approach aiming at preserving the binaural cues or the IC of the diffuse noise component. Contrary to the MVDR, the MVDR-N aims for the output noise component to be equal to a scaled version of the input noise component in the reference microphone signals. It was shown in Cornelis et al. (2010)and Marquardt and Doclo (2018)that the output signals of the MVDR-N are equal to a mixture between the output signals of the MVDR and the (noisy) reference microphone signals.

Similarly as for the MVDR-IC, it was experimentally shown in Marquardt and Doclo (2018)that the MVDR-N preserves the binaural cues of the desired speech component and that a trade-off exists between IC preservation of the diffuse noise component and reduction of the diffuse noise component, which can be controlled by the mixing (trade-off) parameter.

#### Determination of the Trade-Off Parameters

**Figure 2.**
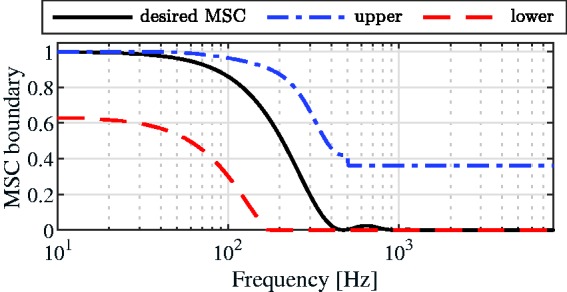
Desired MSC for a Diffuse Noise Field and Psycho-Acoustically Optimized Lower and Upper MSC Boundaries. MSC = magnitude squared coherence.

For the MVDR-IC and the MVDR-N algorithms, a trade-off between IC preservation of the diffuse noise component and reduction of the diffuse noise component, that is, the output SNR, exists. Aiming at an optimal trade-off between noise reduction performance and preserving the spatial impression of a diffuse noise field, in this study, we use psycho-acoustically optimized trade-off parameters that are based on the IC discrimination ability of the (normal-hearing) auditory system for a diffuse noise field. Psycho-acoustical experiments have shown that the perceived width of a sound source mainly depends on the absolute value of the IC (Kurozumi & Ohgushi, 1983). In addition, several IC discrimination experiments have shown that sensitivity to changes from a reference IC strongly depends on the reference IC value (e.g., Culling et al., 2001; Gabriel & Colburn, 1981; Tohyama & Suzuki, 1989; Walther & Faller, 2013). For a reference IC close to 1, small changes can be perceived, whereas for a reference IC close to 0, the auditory system is less sensitive to changes. Based on these experiments, in Marquardt, Hohmann, et al. (2015)it was proposed to impose a constraint on the output MSC of the diffuse noise component by means of frequency-dependent lower and upper MSC boundaries. For frequencies below 500 Hz, these boundaries are a function of the desired MSC for a diffuse noise field, whereas for frequencies above 500 Hz, a fixed lower MSC boundary of 0 and a fixed upper MSC boundary of 0.36 (corresponding to an IC of ±0.6) are used (see Figure 2). Although an upper MSC boundary of 0.36 should lead to an output noise field that cannot be discriminated from a diffuse noise field, we also considered an upper MSC boundary of 0.04 (corresponding to an IC of ±0.2), which leads to even better IC preservation of the diffuse noise component but also to less reduction of the diffuse noise component.

The optimal (frequency-dependent) trade-off parameter in the MVDR-IC yielding the desired output MSC needs to be determined using an iterative procedure (Marquardt, Hohmann, et al., 2015), whereas a closed-form expression exists for the optimal trade-off parameter in the MVDR-N (Marquardt & Doclo, 2018).

These six *algorithm conditions*are summarized in Table 1.

**Table 1.** Summary of the Algorithm Conditions Used in Our Experiment.

Algorithm condition	Description
MVDR	The binaural MVDR beamformer (Doclo et al., 2015), maximizing the output SNR but not preserving the IC of the diffuse noise component.
MVDR-OPT	Artificially generated binaural signals with the same output SNR as the binaural MVDR beamformer but perfectly preserving the IC of the diffuse noise component.
MVDR-IC (0.36) and MVDR-IC (0.04)	The MVDR-IC (Marquardt, Hohmann, et al., 2015) using the upper MSC boundaries for frequencies above 500 Hz of 0.36 and 0.04, respectively.
MVDR-N (0.36) and MVDR-N (0.04)	The MVDR-N (Cornelis et al., 2010; Klasen et al., 2007; Marquardt & Doclo, 2018) using the upper MSC boundaries for frequencies above 500 Hz of 0.36 and 0.04, respectively.

*Note.*MVDR = minimum-variance-distortionless-response; SNR = signal-to-noise ratio; IC = interaural coherence; OPT = optimal.

### Signals and Implementation

All signals were sampled at a sampling frequency of 16 kHz. The binaural algorithms were implemented in the short-time Fourier transform domain using a 16 ms square-root Hann window with 50% overlap.

The speech components in the microphone signals were generated by convolving clean speech material with measured impulse responses for a binaural behind-the-ear hearing aid setup mounted on an artificial head, either in an *anechoic scenario*or a reverberant *cafeteria scenario*with a reverberation time of 1.2 s (Kayser et al., 2009). For each hearing aid, two microphones with an intermicrophone distance of about 7 mm were used. For both acoustic scenarios, the speech source was located in front of the artificial head either at a distance of about 0.8 m (anechoic scenario) or 1 m (cafeteria scenario).

**Figure 3.**
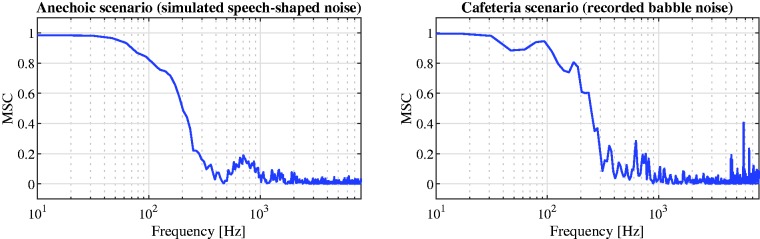
MSC Between the Noise Components in Both Reference Microphone Signals for the Simulated Diffuse Noise Field (Left) and the Recorded Ambient Cafeteria Noise (Right). MSC = magnitude squared coherence.

Two different types of additive noise were used for the experiments. One, for the anechoic scenario, stationary speech-shaped noise was used as the noise signal (Wagener, Brand, et al., 1999b). Using this noise signal, a perfectly diffuse noise field was simulated at the hearing aid microphones using the method described in Habets et al. (2008), such that a desired spatial coherence was obtained. Second, for the cafeteria scenario, a realistic diffuse-like noise field was used, namely, ambient cafeteria noise from Kayser et al. (2009), which was recorded at the hearing aid microphones in a crowded cafeteria at the University of Oldenburg. Besides rather diffuse multitalker babble noise, this noise also contained miscellaneous sounds such as clacking plates and interfering speakers and hence differed from a perfectly diffuse noise field. For both forms of noise fields, Figure 3depicts the (long-term) MSC between the noise components in both reference microphone signals. It can be observed that the MSCs for both noise fields are similar and comparable to the theoretical MSC depicted in Figure 1(right; with head).

For all algorithms, the anechoic ATF vector afrom Kayser et al. (2009)for the frontal direction was used. The (time-invariant) diffuse spatial coherence matrix Γwas calculated using measured anechoic ATFs from Kayser et al. (2009), corresponding to 72 angles between 180° and 175° in steps of 5° on the horizontal plane around an artificial head wearing two behind-the-ear hearing aids (Marquardt & Doclo, 2018). Hence, for all algorithms, the resulting filters were time invariant, and it was *assumed*that the noise component was perfectly diffuse. It should be noted that for the anechoic scenario the same anechoic ATFs and diffuse spatial coherence matrix were used in the algorithms as for generating the speech and diffuse noise components. For this scenario, no estimation errors occurred, and the *optimal performance*for the MVDR, MVDR-IC and MVDR-N was obtained. On the other hand, for the reverberant cafeteria scenario, the assumed diffuse spatial coherence matrix did not exactly match the spatial coherence matrix of the recorded ambient noise and using anechoic ATFs resulted in partial dereverberation of the speech component. For this scenario, estimation errors occurred, and a more *realistic performance*for the MVDR, MVDR-IC, and MVDR-N was obtained. Examples of audio samples for all algorithms and the unprocessed input signals are available online (see https://uol.de/en/sigproc/research/audio-demos/binaural-noise-reduction/ic-preservation-in-binaural-mvdr).

### Measurement Procedures

#### Speech Intelligibility

The SRT was measured using speech material of seven lists from the Oldenburg sentence test (Wagener, Brand, et al., 1999a, 1999b; Wagener, Kühnel, et al., 1999), which is a German matrix sentence test with a male speaker and semantically unpredictable sentences of the fixed syntactical structure *name verb numeral adjective object*, for example, *Kerstin nahm acht schwere Steine*(*Kerstin took eight heavy stones.*). The SRT at 50% speech intelligibility was determined using an adaptive procedure, where the SNR for each presented sentence was adaptively adjusted based on the number of words that were correctly identified in the previous sentence and a convergence factor (Brand & Kollmeier, 2002). The initial SNR was set to 0 dB. Each sentence was preceded by 1 to 2 s of noise-only signal, such that the participants did not exactly know when the sentence started. The participants were instructed to repeat all words of each presented sentence and an instructor checked how many words were correctly understood. Initially, the SNR step size for each iteration is typically rather large depending on the number of words that have been correctly understood (Brand & Kollmeier, 2002). After some iterations, the SNR step size decreases and the SNR converges toward the SRT. To familiarize the participants with the signals and the task (Wagener, Kühnel, et al., 1999), for each participant, two training lists were presented using the unprocessed signals. The first training list was presented at a fixed SNR of 0 dB, which should be easily understandable for normal-hearing participants. The second training list was used to familiarize the participants with the adaptive test procedure. The data from the training lists were discarded.

We conducted two experiments: one for the anechoic scenario and one for the reverberant cafeteria scenario. For each experiment, the SRT was measured for the unprocessed reference microphone signals (UNPROC) and the six algorithm conditions described earlier, resulting in seven conditions in total. For each participant, the order of the conditions, the order of the sentence lists, and the order of the sentences in each list were randomized.

#### Spatial Quality

Spatial quality was evaluated using a procedure similar to the MUlti Stimulus test with Hidden Reference and Anchor (MUSHRA) (International Telecommunications Union-Recommendation BS.1534-1, 2003). In this procedure, all algorithm conditions, including a hidden reference condition and an anchor condition, were compared with a reference condition. All conditions were then rated on a continuous quality scale (0–100) using sliders in a graphical user interface.

In this study, the participants were instructed to rate the overall spatial similarity between the reference condition and each test condition, where a high score corresponded to a small difference to the reference condition and a low score corresponded to a large difference to the reference condition. As all the binaural algorithms are distortionless for the desired speech source, it is expected that all perceived spatial differences can be attributed to the different noise components. Therefore, the participants were instructed to mainly focus on the noise component. The participants were allowed to listen to the reference condition and all test conditions as often as they wanted. For the experienced listeners in our study, it can be assumed that a more precise explanation, for example, in terms of auditory attributes as proposed by Lindau et al. (2014), is not required.

MVDR-OPT was used as the reference condition in this study. In this, the noise component of the MVDR-OPT is equal to a scaled version of the noise component in the unprocessed reference microphone signals with the same SNR as the MVDR, hence combining best noise reduction and perfect IC preservation. Using MVDR-OPT instead of UNPROC as the reference condition should avoid the spatial quality ratings to be dominated by the noise reduction performance of the beamforming algorithms. The hypothesis was to observe low scores (i.e., large spatial difference) for the MVDR, not preserving the IC of the noise component, with the MVDR-IC and the MVDR-N scoring between the MVDR and the MVDR-OPT (reference), as they partially preserve the IC of the noise component. As anchor condition, a monaural signal, obtained by averaging the left and right output signals of the MVDR-OPT, was used. The participants were instructed to rate at least one test condition with a score of 100, which should correspond to the hidden reference. The same acoustic scenarios (anechoic and cafeteria) and the same six algorithm conditions as for the speech intelligibility test were used. As speech material, three sentences from the German matrix sentence test described earlier were concatenated. The intelligibility-weighted SNR (iSNR; Greenberg et al., 1993) of the unprocessed reference microphone signals was set to −5 dB, such that the output iSNR for all algorithm conditions was around 0 dB (see iSNR improvement in Figures 6and 7). The iSNR is defined as the sum of the SNRs in all frequency bins weighted with a frequency-dependent band importance function, for which the same weights as for the speech intelligibility index in American National Standard Institute S3.5-1997 (1997)were used in this study (based on one-third octaves). For a perfectly diffuse noise field, the iSNR improvement is equivalent to the commonly used articulation index-weighted directivity index (AI-DI).

### Participants

In total, 15 self-reported normal-hearing participants (12 male and 3 female participants) with a mean age of 31 (±6.1) years participated in this study. All participants participated in the speech intelligibility test, while 11 of the participants participated in the spatial quality test. All participants were experienced listeners who were familiar with the tasks and the measurement procedures used in this study. The speech intelligibility and the spatial quality test were conducted in separate sessions of about 1 hr on different days. Ethical approval for all experiments was obtained from the ethics committee of the University of Oldenburg. Before the experiments started, informed consent was obtained from all participants.

### Signal Presentation

The measurement tools were implemented on a computer running Microsoft Windows 7 with MATLAB. The binaural signals were presented at 65 dB SPL using an RME Babyface external sound card in combination with Sennheiser HDA 200 headphones. All measurements were performed in an acoustically treated room.

### Statistical Analysis

For the speech intelligibility test and the spatial quality test, a statistical analysis was conducted using the resulting SRT and spatial quality scores, respectively. For each acoustic scenario (anechoic and cafeteria), a one-way repeated-measures analysis of variance (ANOVA) was performed, with factor *algorithm*as dependent variable and factor *participant*as independent variable. If the within-participants effect algorithm was significant, post hoc pairwise comparison *t*-tests with Holm-Bonferroni correction were conducted to test for statistically significant differences between the factor means.

### Objective Measures

To compare the SRT results from the speech intelligibility test to an objective measure, the iSNR improvement (left and right hearing device) of our binaural algorithms compared with the unprocessed reference microphone signals was used. To compare the scores from the spatial quality test to an objective measure, the frequency-averaged MSC error for the diffuse noise component (Marquardt & Doclo, 2018; Marquardt, Hohmann, et al., 2015) between the output signals and the unprocessed reference microphone signals was used. The MSC error was not directly used as an objective measure for spatial quality, but because the expected spatial differences are attributable to the IC of the output noise component (and hence the MSC), the MSC error was used an objective measure for IC preservation of the diffuse noise field.

## Results

### Speech Intelligibility Test

#### Anechoic Scenario

**Figure 4.**
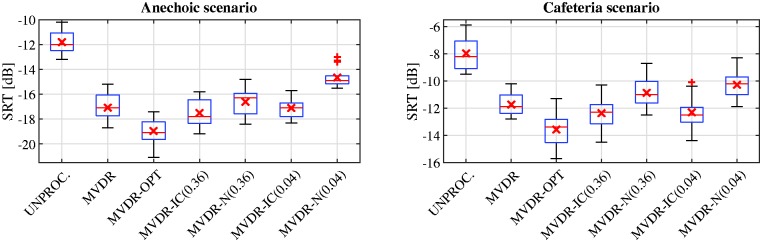
Boxplot of the SRT Results for the Unprocessed Signals and the Evaluated Binaural Algorithms for the Anechoic Scenario (Left) and the Cafeteria Scenario (Right). The boxplot visualizes the interquartile range (IQR) from the 25% percentile to the 75% percentile, and the vertical line inside the box visualizes the median value. The upper whisker indicates the largest value that is smaller than the 75% percentile plus 1.5 times the IQR, and the lower whisker indicates the smallest value that is larger than the 25% percentile minus 1.5 times the IQR. The means are indicated by the cross markers. Outliers are indicated by the + markers. SRT = speech reception threshold; MVDR = minimum-variance-distortionless-response; UNPROC = unprocessed reference microphone signals; IC = interaural coherence; OPT = optimal.

The SRT results for the anechoic scenario are depicted in Figure 4(left). Mauchly’s test, $\chi^{2}$(20) = 16.73, *p* = .684, did not indicate violation of sphericity. The ANOVA demonstrated a significant effect of the factor algorithm, *F*(6, 84) = 353.92, *p* < .001. The mean SRT for the unprocessed signals was equal to −11.8 dB. The mean SRT for the MVDR was equal to −17.1 dB, that is, the mean SRT improvement compared with the unprocessed signals was equal to 5.3 dB. The mean SRT for the MVDR-OPT was further improved to −19.0 dB, that is, the mean SRT improvement compared with the unprocessed signals was equal to 7.2 dB, which is an improvement of 1.9 dB compared with the MVDR. For the practically feasible algorithms MVDR-IC (0.36) and MVDR-IC (0.04), the mean SRT was equal to −17.5 dB and −17.1 dB, respectively. Hence, for both MSC boundaries, the MVDR-IC yielded a similar mean SRT as the MVDR. For the MVDR-N (0.36) and the MVDR-N (0.04), the mean SRT was equal to −16.6 dB and −14.7 dB, respectively. Hence, the impact of the upper MSC boundary on speech intelligibility appeared to be more prominent for the MVDR-N than for the MVDR-IC. Furthermore, for both upper MSC boundaries, the MVDR-IC yielded a better mean SRT than the MVDR-N.

The results of the post hoc tests are given in Table 2. These results show that all algorithms yielded a significant SRT improvement compared with the unprocessed signals and that the MVDR-OPT performed significantly better than all other algorithms. Although the mean SRT for the MVDR-IC (0.36) was better than for the MVDR, this difference was not significant. For the MVDR-N, there was no significant difference between the MVDR-N (0.36) and the MVDR, whereas there was a significant difference between the MVDR-N (0.04) and the MVDR. While the SRT differences between the MVDR-N (0.36) and the MVDR-IC were not significant, the MVDR-N (0.04) performed significantly worse than all other algorithms.

**Table 2.** Significance of the SRT Differences Between Conditions for the Anechoic Scenario.

	UNPROC	MVDR	MVDR-OPT	MVDR-IC (0.36)	MVDR-N (0.36)	MVDR-IC (0.04)	MVDR-N (0.04)
UNPROC		***	***	***	***	***	***
MVDR	***		***	—	—	—	***
MVDR-OPT	***	***		***	***	***	***
MVDR-IC (0.36)	***	—	***		***	—	***
MVDR-N (0.36)	***	—	***	***		—	***
MVDR-IC (0.04)	***	—	***	—	—		***
MVDR-N (0.04)	***	***	***	***	***	***	

*Note.*MVDR = minimum-variance-distortionless-response; IC = interaural coherence; UNPROC = unprocessed reference microphone signals; OPT = optimal.

* *p* < .05. **p < .01. ***p < .001.

#### Cafeteria Scenario

The SRT results for the reverberant cafeteria scenario are depicted in Figure 4(right). Mauchly’s test, $\chi^{2}$(20) = 16.87, *p* = .676, did not indicate violation of sphericity. The ANOVA demonstrated a significant effect of the factor algorithm, *F*(6, 84) = 136.54, *p* < .001. The mean SRT for the unprocessed signals was equal to −8.0 dB, that is, 3.8 dB worse than for the anechoic scenario. The mean SRT for the MVDR was equal to −11.7 dB, that is, the mean SRT improvement compared with the unprocessed signals was equal to 3.7 dB. The mean SRT for the MVDR-OPT was further improved to −13.6 dB. Hence, similarly to the anechoic scenario, perfectly preserving the IC of the diffuse noise component resulted in a mean SRT improvement compared with the MVDR, which was again equal to 1.9 dB. The mean SRT for the MVDR-IC (0.36) and the MVDR-IC (0.04) was equal to −12.4 dB and −12.3 dB, respectively. Hence, for both upper MSC boundaries, a similar mean SRT was obtained, which was about 0.5 dB better than for the MVDR. The mean SRT for the MVDR-N (0.36) and the MVDR-N (0.04) was equal to −10.9 dB and −10.3 dB, respectively. Hence, for both upper MSC boundaries, the mean SRT for the MVDR-N was worse than for the MVDR-IC and the MVDR. In addition, the mean SRT difference between the MVDR-N (0.36) and the MVDR-N (0.04) was smaller for the reverberant cafeteria scenario (0.6 dB) than for the anechoic scenario (1.9 dB).

The results of the post hoc tests are given in Table 3. Similarly to the anechoic scenario, all algorithms yielded a significant SRT improvement compared with the unprocessed signals, and the MVDR-OPT performed significantly better than all other algorithms. Although the mean SRT for the MVDR-IC for both upper MSC boundaries was better than for the MVDR, this difference was only significant for the MVDR-IC(0.36). The MVDR-N for both upper MSC boundaries performed significantly worse than all other algorithms, where the MVDR-N (0.04) performed significantly worse than the MVDR-N (0.36).

**Table 3.** Significance of the SRT Differences Between Conditions for the Cafeteria Scenario.

	UNPROC	MVDR	MVDR-OPT	MVDR-IC (0.36)	MVDR-N (0.36)	MVDR-IC (0.04)	MVDR-N (0.04)
UNPROC		***	***	***	***	***	***
MVDR	***		***	*	***	—	***
MVDR-OPT	***	***		***	***	**	***
MVDR-IC (0.36)	***	*	***		***	—	***
MVDR-N (0.36)	***	***	***	***		***	*
MVDR-IC (0.04)	***	—	**	—	***		***
MVDR-N (0.04)	***	***	***	***	*	***	

*Note.*MVDR = minimum-variance-distortionless-response; IC = interaural coherence; UNPROC = unprocessed reference microphone signals; OPT = optimal.

* *p* < .05. **p < .01. ***p < .001.

In summary, the SRT results for the anechoic and the cafeteria scenario showed that all the binaural noise reduction algorithms were able to significantly improve speech intelligibility. Compared with the MVDR, the MVDR-IC and the MVDR-N partially preserve the IC of the diffuse noise component but degrade the output SNR, where both effects seemed to compensate each other in terms of speech intelligibility. Only the MVDR-IC (0.36) in the cafeteria scenario yielded a small but significant improvement in speech intelligibility, whereas the MVDR-N (0.04) yielded a significant degradation in speech intelligibility for both scenarios. Furthermore, for the MVDR-IC, the upper MSC boundary did not seem to have a significant impact on speech intelligibility, whereas the MVDR-N (0.36) performed significantly better than the MVDR-N (0.04) for both scenarios.

### Spatial Quality Test

#### Anechoic Scenario

**Figure 5.**
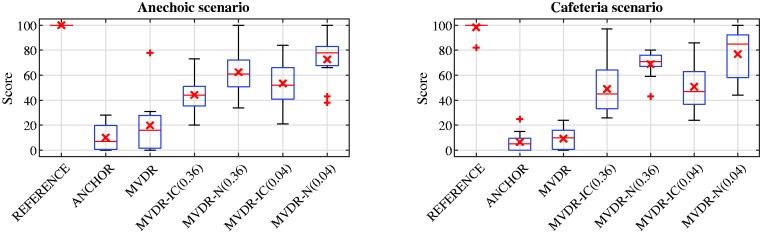
Boxplot of the Spatial Quality Scores for the Evaluated Binaural Algorithms for the Anechoic (Left) and the Cafeteria Scenario (Right). See Figure 4for boxplots details. MVDR = minimum-variance-distortionless-response; IC = interaural coherence.

The results of the spatial quality test for the anechoic scenario are depicted in Figure 5(left), and the results of the post hoc tests are given in Table 4. Because Mauchly’s test, $\chi^{2}$(20) = 38.09, *p* = .013, indicated violation of sphericity, a Greenhouse–Geisser correction was applied. The ANOVA demonstrated a significant effect of the factor algorithm, *F*(2.513, 25.127) = 41.731, *p* < .001. The mean score for the reference condition was equal to 100, showing that the participants were able to distinguish the hidden reference condition from the other test conditions. As desired, the anchor condition achieved the lowest mean score of 10.2. The mean score for the MVDR was equal to 19.9, which was significantly lower than the reference condition and even not significantly higher than the anchor condition. For the MVDR-IC (0.36) and the MVDR-IC (0.04), the mean score was equal to 44.3 and 53.5, respectively. For the MVDR-N (0.36) and the MVDR-N (0.04), the mean score was equal to 62.5 and 72.7, respectively. For both the MVDR-N and the MVDR-IC, the impact of the upper MSC boundary on spatial quality was not significant, and only the difference between the MVDR-N (0.04) and the MVDR-IC (both upper MSC boundaries) was statistically significant. Nevertheless, for both algorithms, the mean scores for an upper MSC boundary of 0.04 were better than for an upper MSC boundary of 0.36 because the former leads to a better IC preservation of the diffuse noise component. Although there was still a significant difference in mean spatial quality scores between the reference condition and the MVDR-IC and MVDR-N, both achieved a significant improvement in terms of spatial quality compared with the MVDR.

**Table 4.** Significance of the Spatial Quality Differences Between Conditions for the Anechoic Scenario.

	Ref.	Anchor	MVDR	MVDR-IC (0.36)	MVDR-N (0.36)	MVDR-IC (0.04)	MVDR-N (0.04)
UNPROC		***	***	***	***	***	**
MVDR	***		—	**	***	***	***
MVDR-OPT	***	—		*	*	*	**
MVDR-IC (0.36)	***	**	*		—	—	*
MVDR-N (0.36)	***	***	*	—		—	—
MVDR-IC (0.04)	***	***	*	—	—		*
MVDR-N (0.04)	**	***	**	*	—	*	

*Note.*MVDR = minimum-variance-distortionless-response; IC = interaural coherence; UNPROC = unprocessed reference microphone signals; OPT = optimal.

* *p* < .05. **p < .01. ***p < .001.

#### Cafeteria Scenario

The results of the spatial quality test for the reverberant cafeteria scenario are depicted in Figure 5(right), and the results of the post hoc tests are given in Table 5. Because Mauchly’s test, $\chi^{2}$(20) = 41.04, *p* = .006, indicated violation of sphericity, a Greenhouse–Geisser correction was applied. The ANOVA demonstrated a significant effect of the factor algorithm, *F*(2.580, 25.798) = 70.214, *p* < .001. Overall, the results were similar to the anechoic scenario. The mean score for the reference condition was equal to 98.4, showing that almost all participants were able to distinguish the hidden reference condition from the other test conditions. In addition, the anchor condition achieved the lowest mean score of 6.5. Similarly to the anechoic scenario, the mean score for the MVDR of 9.3 was significantly lower than for the reference condition and not significantly higher than for the anchor condition. For the MVDR-IC (0.36) and the MVDR-IC (0.04), the mean score was equal to 49.0 and 50.8, respectively. For the MVDR-N (0.36) and the MVDR-N (0.04), the mean score was equal to 68.9 and 76.9, respectively. Similarly to the anechoic scenario, for both upper MSC boundaries, the mean score for the MVDR-N was better than for the MVDR-IC, but the impact of the upper MSC boundary on spatial quality was not significant for both algorithms, and only the difference between the MVDR-N (0.04) and the MVDR-IC (0.04) was statistically significant. Compared with the MVDR, both the MVDR-IC and the MVDR-N achieved a significant improvement in terms of spatial quality. Furthermore, it should be noted that for the cafeteria scenario, there was no statistically significant difference in spatial quality between the reference condition and the MVDR-N (0.04).

**Table 5.** Significance of the Spatial Quality Differences Between Conditions for the Cafeteria Scenario.

	Ref.	Anchor	MVDR	MVDR-IC (0.36)	MVDR-N (0.36)	MVDR-IC (0.04)	MVDR-N (0.04)
UNPROC		***	***	***	***	***	—
MVDR	***		—	***	***	***	***
MVDR-OPT	***	—		**	***	***	***
MVDR-IC (0.36)	***	***	**		—	—	—
MVDR-N (0.36)	***	***	***	—		—	—
MVDR-IC (0.04)	***	***	***	—	—		*
MVDR-N (0.04)	—	***	***	—	—	*	

*Note.*MVDR = minimum-variance-distortionless-response; IC = interaural coherence; UNPROC = unprocessed reference microphone signals; OPT = optimal.

* *p* < .05. **p < .01. ***p < .001.

In summary, from the spatial quality results for the anechoic and the cafeteria scenario, we can conclude that both the MVDR-IC and the MVDR-N were able to significantly improve spatial quality compared with the MVDR. For both scenarios, the MVDR-N achieved higher mean scores than the MVDR-IC, where the choice of the psycho-acoustically motivated upper MSC boundary did not have a statistically significant impact on spatial quality for both algorithms.

### Relation to Objective Measures

**Figure 6.**
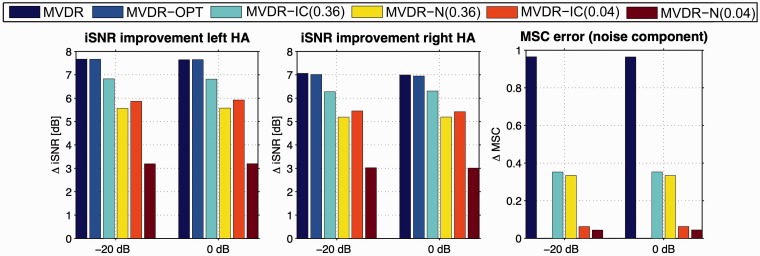
Intelligibility Weighted SNR Improvement in the Left and the Right HA and MSC Error for the Diffuse Noise Component, Averaged Over 20 Sentences for Input iSNRs of −20 dB and 0 dB, for the Anechoic Scenario. MVDR = minimum-variance-distortionless-response; IC = interaural coherence; SNR = signal-to-noise ratio; MSC = magnitude squared coherence; HA = hearing aid; OPT = optimal.

**Figure 7.**
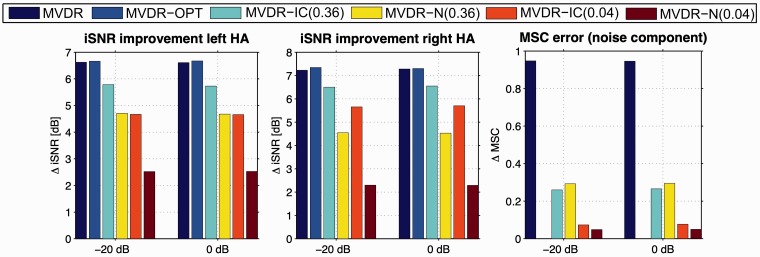
Intelligibility Weighted SNR Improvement in the Left and the Right HA and MSC Error for the Diffuse Noise Component, Averaged Over 20 Sentences for iSNRs of −20 dB and 0 dB, for the Cafeteria Scenario. MVDR = minimum-variance-distortionless-response; IC = interaural coherence; SNR = signal-to-noise ratio; MSC = magnitude squared coherence; HA = hearing aid; OPT = optimal.

For the anechoic scenario and the cafeteria scenario, Figures 6and 7depict the iSNR improvement in the left and the right hearing aid and the MSC error for the diffuse noise component, averaged over 20 sentences, for input iSNRs of −20 dB and 0 dB. As expected, for all the algorithms, both the iSNR improvement and the MSC error were independent of the input SNR, as these algorithms only exploit spatial and no spectral information. For both scenarios, the iSNR improvement for the MVDR-OPT was nearly the same as for the MVDR, whereas the MSC error was very large (close to 1) for the MVDR and equal to 0 for the MVDR-OPT. In addition, the iSNR improvement for the MVDR-IC and the MVDR-N was smaller than for the MVDR, but the MVDR-IC and the MVDR-N also yielded a smaller MSC error than the MVDR. In general, the MSC errors for the MVDR-IC and the MVDR-N were very similar and corresponded to the used MSC boundary. For both upper MSC boundaries, the iSNR improvement for the MVDR-IC was larger than for the MVDR-N, especially for an upper MSC boundary of 0.04.

Comparing the SRT results (Figure 4) to the iSNR results (Figures 6and 7), it can be observed that for the MVDR-OPT, the SRT improvements compared with the unprocessed condition were very similar to the iSNR improvements, while for the MVDR the SRT improvements were smaller than the iSNR improvements. For the MVDR-IC and the MVDR-N, the SRT improvements were also smaller than the iSNR improvements, except for the MVDR-N (0.04), where the SRT improvements and the iSNR improvements are very similar. For algorithms that introduce only a small amount of binaural cue distortion for the diffuse noise component, the iSNR improvement seemed to be a good indicator for the SRT improvement. On the other hand, for algorithms that introduce a large amount of binaural cue distortion, the usage of more advanced models that also take the binaural cues for predicting speech intelligibility into account may be more appropriate (e.g., Beutelmann et al., 2010).

## General Discussion

To assess the influence of the trade-off between noise reduction and IC preservation of the diffuse noise component on speech intelligibility and spatial quality for normal-hearing participants, in this study, we reported perceptual comparisons of several binaural MVDR-based noise reduction algorithms in diffuse noise fields.

### Spatial Release From Masking

The MVDR preserves the binaural cues of the desired speech source but distorts the IC of the diffuse noise component, whereas the artificially generated MVDR-OPT preserves both the binaural cues of the desired speech source and the IC of the diffuse noise component. As both algorithms yield the same output SNR and output speech component, the SRT improvement of 1.9 dB for the MVDR-OPT compared with the MVDR (both for the anechoic and the cafeteria scenario) can be explained solely by the perfect IC preservation of the diffuse noise field. This is consistent with the results in Bronkhorst and Plomp (1992), where the effect of up to six speech-like maskers on speech intelligibility was investigated in a controlled environment. For four maskers, symmetrically placed around the listener, an SRT improvement of about 1.9 dB was reported compared with when all maskers were placed in front of the listener. For six maskers, the SRT improvement was about 1.5 dB. Arweiler and Buchholz (2011)reported a slightly larger SRT improvement up to 3 dB by comparing binaural hearing to monaural hearing for a scenario with one-directional speech source in a diffuse noise field.

For the MVDR-IC and the MVDR-N, which do not perfectly preserve the IC of the diffuse noise component, a similar (albeit smaller) spatial release from masking can be assumed. Whereas the MVDR-IC and the MVDR-N significantly degrade the objective iSNR improvement compared with the MVDR (see Figures 6and 7), the perceptual SRT measurements in Figure 4show a much smaller degradation or even a small improvement. Furthermore, when relating the spatial quality scores in Figure 5to the degree of spatial separation between the desired speech source and the residual noise component for the MVDR-IC/MVDR-N and the MVDR, the explanation of the differences between the iSNR improvements and the SRT results due to spatial release from masking seems even more evident.

In general, the results of this study for diffuse noise fields are in line with the perceptual studies of van den Bogaert et al. (2008, 2009) for (one or more) directional noise sources, that is, also by partially preserving the IC of a diffuse noise field (MVDR-IC/MVDR-N), it is possible to enable significantly better spatial perception compared with the MVDR, while only reducing speech intelligibility in a limited way.

### Hearing Impairment

Even for normal-hearing participants like those studied here, it is difficult to discriminate IC differences when the reference IC is small (e.g., Culling et al., 2001; Gabriel & Colburn, 1981; Tohyama & Suzuki, 1989; Walther & Faller, 2013). The differences between both of the upper MSC boundaries (0.36 and 0.04) in the MVDR-IC and the MVDR-N are expected to be less pronounced for hearing-impaired participants. As reported in Srinivasan et al. (2016)and Whitmer et al. (2012), both age and hearing loss are important factors compromising performance in such discrimination tasks. Furthermore, Goupell and Litovsky (2015)reported that although cochlear implant users can perceive changes in interaural correlation, their poor performance might be a limiting factor for binaural unmasking in realistic scenarios and therefore presumably also for the binaural MVDR-based algorithms investigated in this study. Further research is needed to investigate the potential of IC preservation of the diffuse noise component for different types of hearing impairment.

### Real-World Application

To compute the filter vectors of the MVDR, two design parameters have to be modeled or estimated: the spatial coherence matrix of the undesired component Γand the ATF vector of the desired speech source a.

In this study, we assumed a diffuse noise field and used anechoic impulse responses on the horizontal plane provided by Kayser et al. (2009)to model the spatial coherence of the diffuse noise components in the microphone signals. As would be expected, our MVDR showed best performance whether the tested diffuse noise field matched the type of diffuse noise field upon which it was designed. In the anechoic scenario, where the diffuse noise field was simulated using the method described in Habets et al. (2008)with the same spatial coherence matrix Γas used in the MVDR, the spatial characteristics of the tested diffuse noise field exactly matched the modeled spatial coherence matrix Γ. This is quite unrealistic in practice but could be seen as a benchmark of the optimal algorithm performance. For the anechoic scenario, the MVDR showed an SRT improvement of 5.3 dB, whereas Völker et al. (2015)reported SRT improvements between 3 and 4.3 dB for several noise scenarios. In the cafeteria scenario, however, the spatial characteristics of the tested diffuse noise field differed from the modeled spatial coherence matrix Γ, especially due to dominant interfering speakers and transient noises such as clacking plates. This scenario can be seen as a benchmark for a realistic diffuse noise field, such that the performance of all the algorithms dropped by about 1 dB compared with the controlled anechoic scenario. A similar performance to the cafeteria scenario can be expected for other realistic diffuse noise fields, for example, in a crowded restaurant or a train station.

Furthermore, the binaural MVDR-based algorithms require the ATFs of the desired speech source. In this study, we assumed that the desired speech source is located in front of the hearing device user and hence selected the ATFs corresponding to the frontal direction from the database with measured ATFs in Kayser et al. (2009). While this is a very common assumption, in practice, the desired speech source is obviously not always located in front of the hearing device user. In this case, the direction-of-arrival (DOA) of the desired speech source needs to be estimated, for which several procedures have been proposed (e.g., Chakrabarty & Habets, 2017; Kayser & Anemüller, 2014; Woodruff & Wang, 2012) and the ATFs corresponding to the estimated DOA should be selected. The influence of DOA estimation errors on the performance of the MVDR was analyzed in Marquardt and Doclo (2016), where it was shown that the noise reduction performance is significantly reduced when DOA estimation errors larger than 10° occur. In addition, it is well known that head-related transfer functions vary significantly from person to person (Algazi et al., 2001). The influence of using personalized ATFs instead of generic ATFs measured on a dummy head, as in Kayser et al. (2009), was investigated in Moore et al. (2019), where it was shown that the additional SRT improvement achieved by using personalized ATFs was only about 0.4 dB for the MVDR.

## Conclusions

In this study with normal-hearing participants, we reported perceptual comparisons of several binaural MVDR-based noise reduction algorithms in diffuse noise fields in order to assess the influence of the trade-off between noise reduction and IC preservation of the diffuse noise component on speech intelligibility and spatial quality.

In terms of speech intelligibility, the results showed that all the binaural noise reduction algorithms resulted in a significant SRT improvement compared with the unprocessed signals. The SRT results for the artificially generated MVDR-OPT indicated that the SRT was improved by around 2 dB compared with the MVDR when the IC of a diffuse noise field was perfectly preserved. As the MVDR-IC and the MVDR-N partially preserve the IC of a diffuse noise field but degrade the output SNR compared with the MVDR, both effects seemed to compensate each other in terms of speech intelligibility. Only for the MVDR-IC (0.36) in the reverberant cafeteria, scenario was a small but significant increase in speech intelligibility obtained compared with the MVDR.

In terms of spatial quality, the results showed that the MVDR-IC and the MVDR-N were able to achieve a significant improvement compared with the MVDR for both of the upper MSC boundaries. While the MVDR-IC achieved a better performance in terms of speech intelligibility compared with the MVDR-N, the MVDR-N showed a better performance in terms of spatial quality, even though the MSC error of the diffuse noise component was very similar for both algorithms.

In summary, this study with normal-hearing participants showed that partially preserving the IC of a diffuse noise field, either by using the MVDR-IC or the MVDR-N, significantly improves spatial quality compared with the MVDR while only marginally affecting speech intelligibility.
